# Nasopharyngeal amyloidoma: report of three cases and review of the literature

**DOI:** 10.1007/s00432-024-05873-5

**Published:** 2024-07-06

**Authors:** Wangsheng Zuo, Yu Du, Jian-ning Chen

**Affiliations:** Present Address: Department of Pathology, The Third Affiliated Hospital, Sun Yat-Sen University, No. 600 Tianhe Road, Guangzhou, 510630 China

**Keywords:** Amyloidosis, Nasopharyngeal, Localized amyloid, Congo red

## Abstract

**Background:**

Nasopharyngeal amyloidoma is a rare, locally aggressive tumor that has been reported in the English literature in only 38 cases to date, most of which were in the form of case reports. The present study was aimed to summarize the characteristics of this rare tumor, with the goal of providing new insights for diagnosis and treatment.

**Materials and methods:**

We report three cases of nasopharyngeal amyloidoma diagnosed in our hospital following comprehensive medical examination and review the current literature on all cases of nasopharyngeal amyloidoma from PubMed. The journey of nasopharyngeal amyloidoma, including presentation, diagnostics, surgeries, and follow-up was outlined.

**Results:**

None of the three patients had systemic amyloidosis. CT and nasal endoscopy showed irregular masses obstructing the nasopharyngeal cavity. Congo red staining confirmed the deposition of amyloid, and immunohistochemical analysis showed that the amyloid deposition was the AL light chain type. Through literature review, we found that nasopharyngeal amyloidoma most commonly occurred in individuals over the age of 40, patients usually had a good prognosis after complete tumor resection; however, there were still cases of recurrence, and unresected patients were at risk of progression to systemic amyloidosis. The efficacy of radiotherapy and chemotherapy was currently uncertain.

**Conclusion:**

Early clinical and pathological diagnosis is crucial, and surgical intervention is the primary treatment option for this disease. Although patients usually have a favorable prognosis, long-term monitoring is necessary to detect potential relapses and initiate timely intervention.

## Introduction

Amyloidosis is caused by the deposition of amyloid protein, which is made up of many different amyloid fibril (Wisniowski and Wechalekar 2020). Amyloid fibrils are non-branching, have a smooth surface, and exhibit exceptional physical and chemical stability. They are capable of binding both dyes, Congo red and thioflavin T (Goldsbury et al. 2011). X-ray diffraction analysis revealed that the fibrillation was organized in a β-pleated structure, and the direction of the polypeptide main chain was perpendicular to the fibril axis (Álvarez-Marimon et al. 2021). According to transmission electron microscopy, the fibrils exhibited a width of 60–130 angstroms (Å) and a length of 100–16000 angstroms (Å) (Sipe and Cohen 2000).

After hematoxylin–eosin staining (HE), amyloid substance is characterized by eosinophilic, amorphous, and homogeneous pink deposits under the skin or around blood vessels and fat, as observed under a microscope (Picken 2020). In 1923, Bennhold discovered that Congo red could stain amyloid, resulting in a brick-red appearance and a characteristic double-refraction apple green color under polarized light filter (Elghetany et al. 1989). This criterion has become crucial for the accurate diagnosis of amyloidosis.

Amyloid deposition can occur in multiple systems throughout the body or in a particular organ. There are over 15 types of amyloidosis, with the most prevalent being light chain (AL) type amyloidosis, which is the most common form of systemic amyloidosis. It is caused by the deposition of immunoglobulin produced by abnormal clonal plasma cells (Deshayes and Aouba 2021). It commonly affects patients with multiple myeloma and primary amyloidosis (Wechalekar et al. 2016). AL amyloidosis accounts for about 70% of all systemic cases of amyloidosis (Rekhtina and Khyshova 2023). The disease progresses rapidly, frequently affecting the heart, liver, skin, and kidneys, and carries a grim prognosis (Hazenberg 2013). If amyloidosis is present at a specific site and forms a tumor-like lesion at that site, it is referred to as amyloidoma (Yerasi et al. 2022).

Nasopharyngeal amyloidoma is an extremely rare disease, and the cause and pathogenesis of the disease remain unclear (Wu et al. 2011). The disease is characterized by the deposition of abnormal, fibrous beta-pleated structures comprised of protein in the nasopharynx, leading to mass formation, and subsequent structural and functional abnormalities in this region. The clinical manifestations of nasopharyngeal amyloidoma are diverse and nonspecific, posing a challenge to its accurate diagnosis.

In this study, we retrospectively analyzed the imaging, clinical, and pathological data of three patients with nasopharyngeal amyloidoma who were diagnosed and treated in the Third Affiliated Hospital of Sun Yat-sen University, Guangzhou, southern China, and all English literature on nasopharyngeal amyloidoma to date was reviewed. Our aim was to provide new insights for the accurate diagnosis of this rare disease and to offer different ideas for its evaluation and treatment.

## Materials and methods

We conducted a retrospective analysis of three patients in our hospital, reviewing their surgical pathology, examination indexes, clinical images, and medical records. All retrieved slides were reviewed, and clinical data were obtained from a comprehensive electronic medical record. In addition, immunohistochemical (IHC) staining of κ (DAKO; A0191) and λ-light (DAKO; A0193) chain were performed. A comprehensive review of the literature was conducted through electronic searches of PubMed using the search terms “nasopharynx” or "nasopharyngeal" and "amyloidosis". Only articles and reviews written in English were included.

## Results

**Fig. 1:**
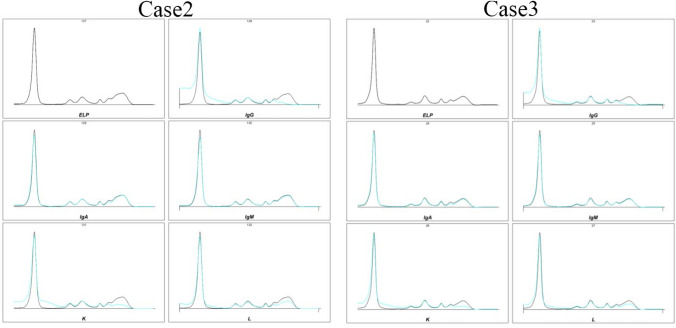
Serum immunofixation electrophoresis was performed on Cases 2 and 3. In both cases, monoclonal immunoglobulins were not detected

In this study, we found that all three patients were over 60 years old and the main complaints were nasal congestion and a runny nose. Blood routine and blood biochemical tests were not abnormal, liver function and renal function tests were within the normal range, and urine routine detection did not reveal any urine protein or ketone bodies (Table 1). The κ/λ ratio in the serum and urine of patients was within the normal range. Additionally, monoclonal immunoglobulins were not detected in the serum immunofixation electrophoresis of two patients (Cases 2 and 3, Fig. 1), while Case 1 was not tested. Ultrasonic examination of the three patients revealed no specific changes in the liver, kidney, heart, gallbladder, pancreas, spleen, and other important organs. Their medical histories also showed no significant problems, indicating that none of them had systemic amyloidosis.

**Table 1:** Laboratory results and basic information of patients

	Reference range	Case 1	Case 2	Case 3
Age (year)		64	79	60
Gender (F/M)		M	F	M
CBC				
WBC (× 10^9^/L)	3.5–9.5	6.49	6.95	8.87
RBC (× 10^12^/L)	4.3–5.8	4.62	5.87	3.72
Platelets (× 10^9^/L)	100–350	183	290	230
Hemoglobin (g/L)	130–175	138	**119**	**112**
CMP				
AST(U/L)	15–40	23	16	20
ALT(U/L)	3–35	17	17	13
Total protein(g/L)	61–82	63.8	67.6	66.8
Albumin(g/L)	36–51	43.4	38.8	35.4
K^+^(mmol/L)	3.5–5.3	4.13	4.25	3.75
Na^+^ (mmol/L)	137–147	143	143	144
Cl^−^ (mmol/L)	99–110	105.8	104.9	107.5
HCO_3_^−^ (mmol/L)	20.2–29.2	23.2	26.5	**29.3**
Glucose (mmol/L)	3.9–6.1	**9.26**	**6.97**	**7.71**
BUN (mmol/L)	2.4–8.2	3.67	5.91	**8.96**
Creatinine (umol/L)	31.8–116	62	57	**136**
Ca2 + (mmol/L)	2.03–2.65	2.34	2.4	2.35
Egfr-EPI		97.4	84.94	93.3
Routine Urine				
GLU	–	–	–	–
PRO	–	–	–	–
WBC	–	–	–	–
KET	–	–	–	–
Kappa in plasma (g/L)	1.7–3.7	2.91	2.85	**4.72**
Lambda in plasma (g/L)	0.9–2.1	1.3	1.88	1.87
The κ/λ ratio in plasma	1.35–2.65	2.24	1.52	2.52
Kappa in urine (mg/L)	0–8	**16**	**26**	**32.4**
Lambda in urine (mg/L)	0–4	**7**	**6.65**	**9.01**
The κ/λ ratio in urine	0.75–4.5	2.29	3.91	3.60

Bold values indicate abnormal results

*CBC* complete blood count, *CMP* complete metabolic panel, *PRO* protein, *KET* ketone body

**Fig. 2:**
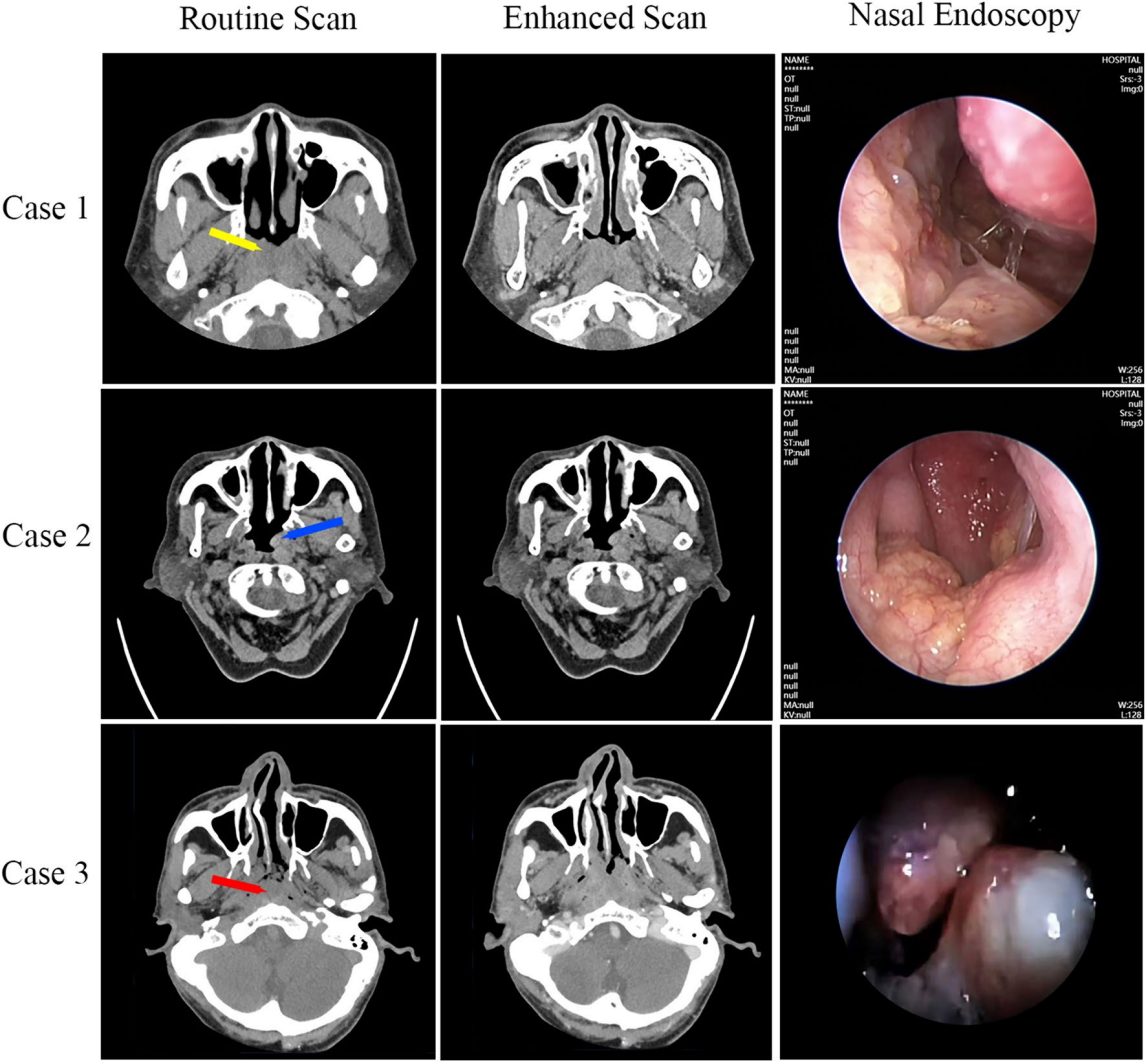
Routine scan, enhanced CT and nasal endoscopy images of three patients. The CT scan revealed low-density, flaky shadows in the nasopharyngeal cavity, with uneven density and an unclear boundary. The enhanced scan displayed mild enhancement and partial non-enhancement. The nasal endoscopy revealed an irregular, gray-red mass obstructing the nasopharyngeal cavity, accompanied by a rough mucosal surface

CT and nasal endoscopy were conducted in all three patients (Fig. 2). Case 1 revealed an irregularly shaped grayish-red mass obstructing the nasopharyngeal cavity. Case 2 discovered a new organism in the right posterior segment of the nasal base and the left lateral wall of the nasopharynx. The organism exhibited a grayish-red or grayish-yellow color, slightly uneven mucosa, and local vasodilation. The CT scan displayed an irregular soft tissue density image with a size of approximately 16 mm × 11 mm and an involved length of about 39 mm. Case 3 found a new organism in the left posterior nostril of the nasal cavity with a smooth surface, light yellow appearance, thickened nasopharyngeal mucosa, and a low-density laminar nasopharyngeal cavity. The lesion extended to the epiglotic region downward, exhibiting uneven density and an unclear boundary. The CT value was approximately 45HU. The enhanced scan showed uneven enhancement in all cases. Notably, no cervical lymph node enlargement was observed in any of the three patients. We observed that the nasopharyngeal mass did not have any significant impact on ventilation.

**Fig. 3:**
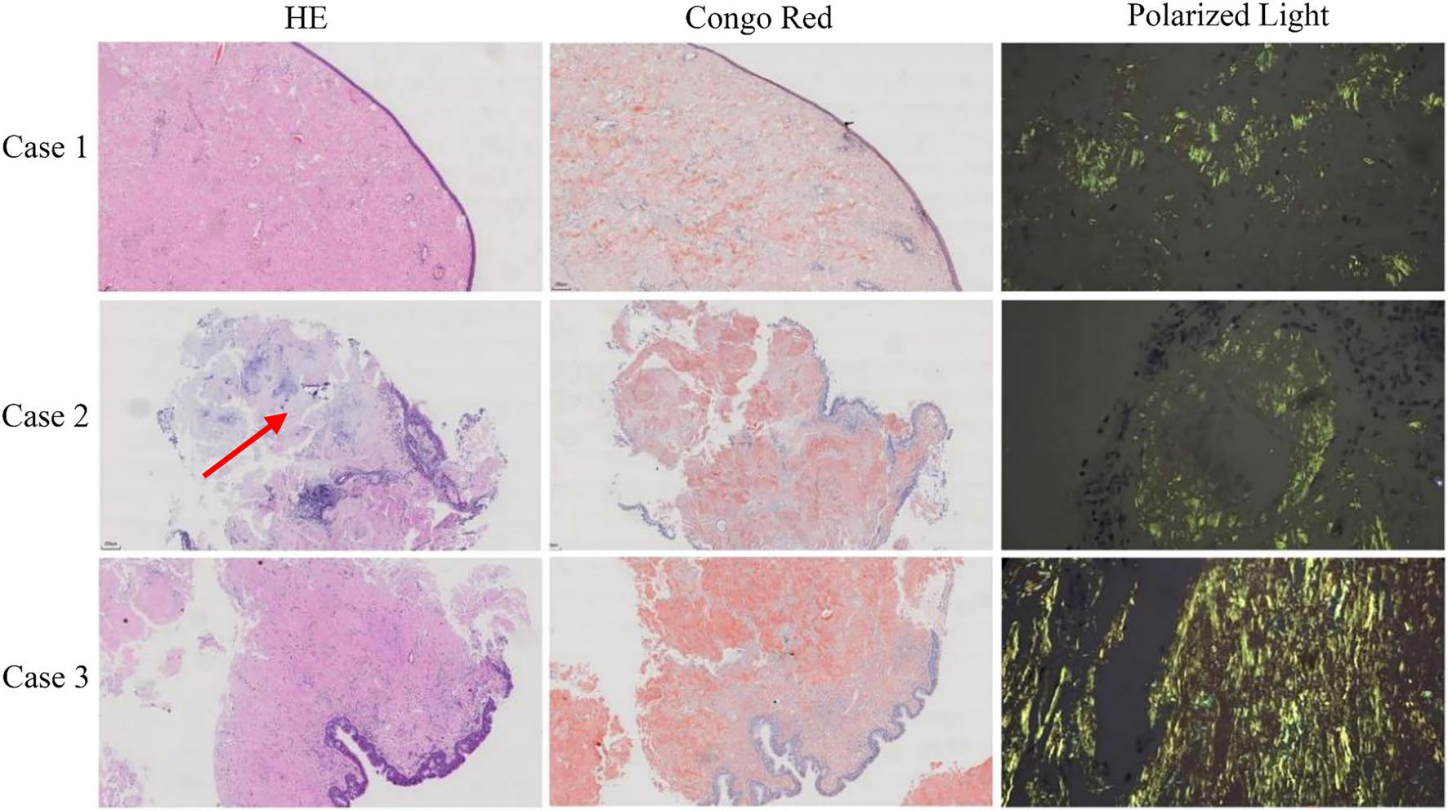
HE and Congo red staining of amyloidoma. Under a light microscope (4 ×), amyloid appears as a uniform pink amorphous substance, the same areas exhibit Congo red staining, resulting in a typical brick-red appearance color. Under a polarized light microscope, Congo red-stained slides exhibit a characteristic birefringent apple green color. Additionally, Case 2 demonstrates local cartilage hyperplasia and calcification, as indicated by the red arrow

**Fig. 4:**
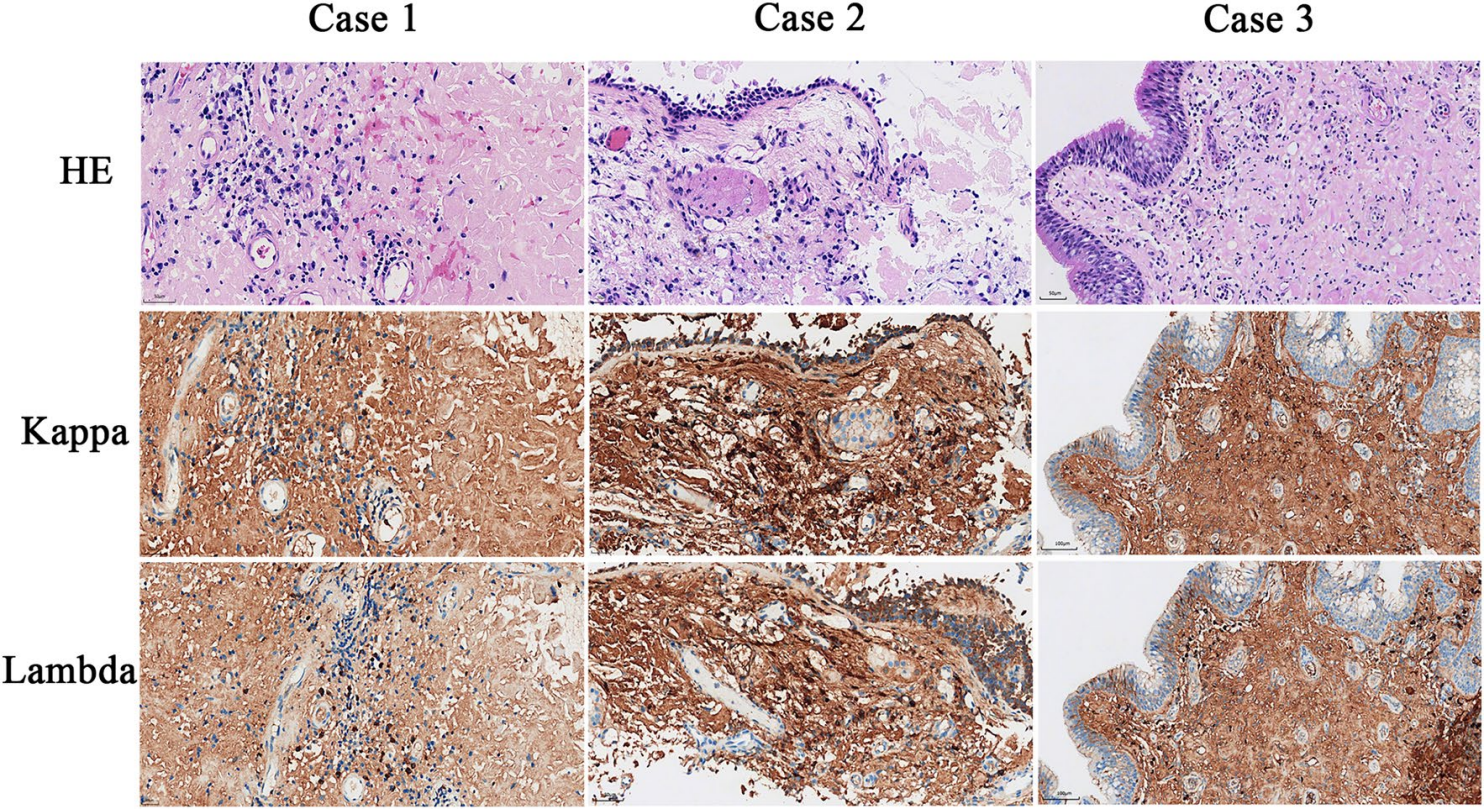
The IHC results of amyloid proteins in 3 cases. IHC staining showing κ and λ light chains immunoglobulin expression in amyloid material and nearby plasma cells (200 ×)

Two of the three patients (Cases 1 and 3) underwent endoscopic plasma resection of oropharyngeal masses via a combined oral-nasal approach. Case 2 was discharged after a small tissue biopsy under nasal endoscopy. The surgical specimens had a maximum diameter of 4.5 cm and 5 cm, respectively, and showed gray-red and yellow colors. All gross specimens were stained with HE and Congo red (Fig. 3). Cases 1 and 3 were mostly polypoid masses covered by squamous epithelium or ciliated columnar epithelium. Amyloid deposition was observed under the epithelium, distributed in small thin-walled blood vessels, accompanied by a few lymphocytes, plasma cells, and a few multinucleated giant cells. The Congo red stained sections show a characteristic uniform brick red color under the microscope and typical apple green birefringence under polarized light microscopy (Fig. 3). Based on the clinical history and Congo red staining results, these findings are consistent with the diagnosis of primary nasopharyngeal amyloidoma. Case 2 was also diagnosed with nasopharyngeal amyloidoma, but presented with focal cartilage hyperplasia and calcification. In addition, IHC staining revealed strong expression of both κ and λ-light chain (Fig. 4). These findings suggested that the amyloid deposited in the three patients was AL, with mixed expression of the κ and λ-light chains.

Following surgical intervention, two patients received symptomatic treatment, including anti-infection medications, nasal drops, and local nasal sprays. The nasal congestion improved significantly, and there was no bloody fluid observed in either anterior nostril. The patients were discharged without any additional discomfort. We followed up the 3 cases for 4 years, 2 months and 1 month, respectively, and showed no recurrence or progression.

### Review of the literature

To date, a total of 38 cases of nasopharyngeal amyloidoma have been reported in the English medical literature. Table 2 provides a summary of the clinical characteristics of nasopharyngeal amyloidoma, including the previously reported 38 cases and our 3 cases. A total of 22 males and 19 females were included in the study. The age of onset ranged from 7 to 86 years (with a mean of 55 years and a median of 60 years), and 33 patients were older than 40 years. Notably, nasopharyngeal amyloidoma typically affects middle-aged and elderly individuals over the age of 40, with no significant difference in gender distribution.

**Table 2:** Clinical and pathological features of nasopharyngeal amyloidoma

No.	Authors and citation	Age -Gender	Clinical Findings and symptoms	Systemic amyloidosis	Greatest dimension (cm)	Extension	Types of amyloid proteins	Treatment	Follow-up	Recurrence or progression
1	Luo et al. (2016)	39-M	NC and epistaxis	NO	4	Nasopharynx	Not stated	Resection and radiotherapy	1 year	NO
2	Patel et al. (2002)	68-M	Decreased hearing	NO	Not stated	Eustachian tube	Not stated	Untreated	3 months	Not stated
3	Jamarun and Wong (2022)	72-M	NC	NO	Not stated	Nasopharynx	Not stated	Resection	1 year	NO
4	Feng et al. (2022)	51-M	Hoarseness	NO	1.2	Nasopharynx and vocal cord	Not stated	Resection	6 months	NO
5	George et al. (2022)	60-M	NC and hearing loss	NO	5	Nasopharynx	Not stated	Not stated	Not stated	Not stated
6	Chen et al.(Chen et al. 2010)	86-M	Intermittent postnasal drip	NO	Not stated	Nasopharynx	Not stated	Untreated	3 years	NO
7	Crosetti and Manca (2021)	80-F	NC	NO	4.3	Nasopharynx	Not stated	Resection	2 years	NO
8	Durbec et al. (2012)	59-F	NC	NO	Not stated	Nasopharynx	Not stated	Resection	5 months	NO
9	Chowsilpa, et al. )^2018^)	64-M	Aural fullness with autophony	NO	3.2	Eustachian tube	Not stated	Resection	3 years	Recurrence and progression to systemic amyloidosis
10	Kim and Kwon )^2017^)	73-M	Frequent epistaxis	NO	Not stated	Nasopharynx	Not stated	Untreated	1 year	NO
11	Wahid et al. (2019)	61-M	NC and recurrent epistaxis	NO	Not stated	Nasopharynx and maxillary sinus	Not stated	Resection	6 months	NO
12	Karimi and Chheda (2010)	56-M	Otalgia and right aural fullness	NO	Not stated	Nasopharynx	Not stated	Untreated	Not stated	Progression to systemic amyloidosis
13	Chen and Sun (2019)	41-F	NC and loss of sense of smell	YES	3	Nasopharynx and rosenmuller fossa	AA	Resection and Chemotherapy	6 months	NO
14	Simpson et al. (1984)	29-F	Not stated	Not stated	Not stated	Nasopharynx and soft palate	Not stated	Laser excision	3 years	NO
15	Hall et al. (2022)	61-F	Constant postnasal drip	NO	Not stated	Nasopharynx	AL	Radiotherapy	3 months	NO
16	Lim et al. (1999)	42-F	NC	NO	Not stated	Nasopharynx	AL	Resection	1 year	NO
17	Sakagiannis et al. (2018)	74-F	NC	NO	Not stated	Nasopharynx and eustachian tube	AA	Resection	2 years	Not stated
18	Sakagiannis et al. (2018)	54-F	NC	NO	Not stated	Nasopharynx	AA	Untreated	29 years	NO
19	Sakagiannis et al. (2018)	7-F	NC	NO	1.1	Nasopharynx	AA	Resection	35 years	NO
20	Georgios et al.(Sakagiannis et al. 2018)	18-F	Difficulty swallowing	NO	3	Nasopharynx	AA	Resection	34 years	NO
21	Sakagiannis et al. (2018)	13-M	Episodes of otitis media	NO	1.1	Nasopharynx	AA	Resection	4 years	NO
22	Sakagiannis et al. (2018)	69-M	Postnasal drip	NO	1.4	Nasopharynx	AA	Not stated	4 years	NO
23	Sakagiannis et al. (2018)	81-F	Chronic rhinosinusitis	Not stated	2.8	Nasopharynx and eustachian tube	AA	Resection	3 months	Recurrence
24	Gomes et al. (2023)	72-F	NC	NO	Not stated	Nasal cavity and pharyngeal region	Not stated	Untreated	Not stated	Not stated
25	Panda et al. (2007)	43-M	Not stated	NO	4.2	Nasopharynx and oropharynx	Not stated	Resection	6 months	NO
26	Geller et al. (2011)	62-F	Bilateral epiphora	NO	Not stated	Nasal cavities and the subglottic tracheal wall	Not stated	Not stated	Not stated	Not stated
27	Susanibar-Adaniya et al. (2017)	44-M	NC and anosmia	YES	9	Nasal cavities and maxillary sinus	AL (κ)	Resection and Chemotherapy	Not stated	NO
28	Wu et al. (2011)	72-M	Dysphagia	NO	6	Orbit and nasopharynx	AL	Not stated	Not stated	Not stated
29	Domínguez et al. (1996)	13-F	NC	NO	Not stated	Nasopharynx	Not stated	Resection	9 months	NO
30	Kumar et al. (2016)	55-M	NC and recurrent nasal bleed	NO	Not stated	Nasopharynx and skull base	AL (κ > λ)	Resection	Not stated	NO
31	Mirza et al. (2013)	31-F	Fluctuating hearing loss	NO	2.3	Nasopharynx and eustachian tube	Not stated	Untreated	2 years	The growth had extended beyond resectable limits
32	Zhuang et al. (2005)	81-F	Neck masses	NO	2	Nasopharynx	Not stated	Resection	9 months	NO
33	Motosugi et al. (2007)	46-F	NC	Not stated	3	Nasopharynx	Not stated	Not stated	Not stated	Not stated
34	Cheng et al. (2009)	60-M	Stuffy nose with hoarse voice	NO	Not stated	Nasal cavity, nasopharynx and larynx	Not stated	Resection	7 months	NO
35	Hegarty and Rao (1993)	77-M	Facial paresis, deafness, dysphagia, hoarseness	NO	Not stated	Nasopharynx and skull base,	AL	Untreated	Not stated	Not stated
36	Pitkäranta and Malmberg (2000)	14-M	Rhinorrhea and right ear obstruction	NO	Not stated	Nasopharynx and tonsil	Not stated	Resection	16 years	Recurrence
37	Pitkäranta and Malmberg (2000)	41-F	Continuous postnasal drip and stertor	NO	2	Nasopharynx	Not stated	Resection	4 years	NO
38	Panda et al. (1994)	82-M	Recurring bleeding from the oral cavity	NO	Not stated	Nasopharynx	Not stated	Resection	1 year	NO
39	Our case	64-M	NC	NO	4.5	Nasopharyngeal cavity	AL	Resection	4 years	NO
40	Our case	79-F	NC	NO	4	Nasopharyngeal	AL	Untreated	2 months	NO
41	Our case	60-M	NC and a runny nose	NO	5	Nasopharyngeal and epiglotic region	AL	Resection	1 month	NO

*M* male, *F* female, *R* right side, *L* left side, *NC* nasal congestion, *AL* light chain amyloidosis, *AA* AA amyloidosis.

Among the 41 cases, approximately half of the patients reported symptoms of nasal obstruction, while the remaining symptoms included epistaxis, persistent rhinorrhea, hearing loss, hoarseness, anosmia, dysphagia, etc. Radiological and nasal endoscopic examinations revealed that the tumors were predominantly located in the nasopharynx, which could involve the nasal cavity, oropharynx, eustachian tube, vocal cords, tonsils, maxillary sinus, cranial base, and orbit.

Macroscopically, the tumors varied in size from 0.1 to 9 cm, with an average size of 3.4 cm and a median size of 3 cm. The cut surfaces were predominantly solid, appearing gray-red to grayish-yellow in color. Histologically, they were characterized by a homogenous, eosinophilic, amorphous material, with occasional lymphocytic infiltration around. Congo red staining revealed a characteristic uniform brick red color under the microscope and apple-green birefringence after polarizing light filtration. Out of the 41 cases, 9 patients had AL type amyloidosis, 8 patients had AA amyloidosis, and 24 patients did not specify the type of amyloid.

Twenty-three patients underwent total tumor resection, which included surgical resection and laser resection, with follow-up ranging from 1 month to 35 years. Of these, 19 patients experienced no recurrence or progression, 3 patients relapsed, and 1 patient's status was not stated. Among the patients who experienced recurrence, 1 patient developed systemic amyloidosis at the 1-year follow-up, 1 patient recurred after 3 months due to incomplete resection of the mass, and 1 patient recurred at the 16-year follow-up after a complete resection of the mass.

Two patients received treatment with excision and systemic chemotherapy, while one patient received excision and radiotherapy. All three patients were followed up for 6 months to 1 year without recurrence or tumor progression. One patient received radiotherapy alone, and after three months of follow-up, the mass did not increase or progress. Nine patients did not receive any treatment, including 1 patient whose tumor progressed too rapidly for follow-up surgery, 1 patient who developed systemic amyloidosis, 5 patients who showed no progression for 3 months to 3 years, and the remaining 2 patients for whom no follow-up information was provided. Treatment and follow-up information were not provided for the remaining 5 patients.

## Discussion

The amyloid proteins can be derived from serum proteins or produced locally, are unable to be metabolically cleared, leading to cell death at the corresponding site, destruction of tissue structure and function, subsequent organ function impairment, and ultimately, related organ dysfunction (Riehani and Soubani 2023). Nasopharyngeal amyloidoma is a distinct entity characterized by the presence of amyloid deposition within the nasopharynx, without evidence of systemic amyloidosis (Desai et al. 2021). Although extremely rare, it is essential to differentiate it from other nasopharyngeal lesions.

In our study, we presented three cases of nasopharyngeal amyloidomas and comprehensively summarized the clinical and pathological information of all reported cases. Nasopharyngeal amyloidoma is a benign tumor that can manifest as single or multiple nodules, gradually invading the surrounding soft tissues from the nasopharynx. Microscopically, under hematoxylin–eosin staining, the tissue appears as a pink, uniform amorphous substance, indicating the presence of protein fibrils that cannot be metabolically cleared. Case 2 exhibited focal chondroplasia and calcification, which may represent a repair response of original fibroblasts in fibrous connective tissue damaged by nonfunctional protein precursors. In addition, IHC staining revealed that the amyloid deposited in the three patients was AL, with mixed expression of the κ and λ-light chains.

Generally speaking, AL amyloidosis is characterized by the presence of a monoclonal light chain, either κ or λ. However, some studies showed that there were mixed presence of both κ and λ light chains in AL amyloidosis(Kumar et al. 2016; Jamshidi et al. 2022). Mass spectrometry can be employed to quantitatively analyze all amyloidosis precursor proteins in tissues with up to 90% sensitivity and specificity in determining the type of amyloidosis. Using mass spectrometry, Jamshidi P et al. reported that λ light chain restriction is the most common mode (6/10, 60%), and the second most common mode is a mixture of λ and κ light chains (3/10, 30%)(Jamshidi et al. 2022). Kumar B et al. also identify mixed expressions of λ and κ light chains in a case of nasopharyngeal amyloidoma (Table 2, Case 30) (Kumar et al. 2016). In our study, all three cases of nasopharyngeal amyloidoma strongly expressed both λ and κ light chains. We acknowledge that IHC staining is not precise for quantifying light chains, detection of both κ and λ light chains in our cases maybe resulted from methodological problems of IHC staining with antibodies against immunogloblins, such as cross-reaction with non-target antigens or non-specific staining. However, due to the significantly higher cost of mass spectrometry compared to IHC, we did not perform mass spectrometry for a more accurate quantification. This is a limitation for the present study.

In clinical practice, histopathology and Congo red staining results are commonly utilized for the diagnosis of tumors, but high-resolution computed tomography evaluation is also a valuable tool as it allows for the assessment of tumor invasion and identification of other nasopharyngeal tumors (Gandhi et al. 2003). The diagnosis of amyloidoma does not rest solely on a single discipline. Rather, it necessitates a comprehensive approach involving a series of laboratory examinations and multidisciplinary consultations, alongside clinical evaluations and laboratory testing. The objective is to exclude systemic amyloidosis and establish a precise diagnosis and appropriate treatment. Here are the appropriate laboratory tests that we recommend to evaluate patients for systemic amyloidosis, including:

1. Serum k chain, λ chain, and k/λ ratio within the normal range, without the presence of autoimmune antibodies (RF, ANA, aCCP anti-C-ANCA antibodies, anti-P-ANCA antibodies).
Negative results from serum immunofixation electrophoresis for monoclonal immunoglobulins.
No evidence of liver or kidney dysfunction.
Urinary protein, k chain, λ chain, and k/λ ratio within the normal range.
Cardiac MRI to exclude cardiac amyloidosis.

Through literature review, we have learned that nasopharyngeal amyloidoma is a benign disease, but its course typically presents as a chronic and aggressive mass, resulting in clinical symptoms such as nasal congestion and dysphagia. Early surgical resection of the complete mass can yield significant benefits. However, in the small number of patients who relapse after resection, we have found that incomplete resection of the mass may lead to recurrence in the short term, and even after complete resection, there are still cases of recurrence during long-term follow-up. Furthermore, there is a possibility that the disease may progress to systemic amyloidosis.

Although no tumor recurrence or progression was observed in the three patients who underwent surgical resection combined with chemotherapy or radiotherapy during the 6-month to 1-year follow-up period, the prognostic effect of surgical combined chemotherapy or radiotherapy remains unclear due to the limited number of cases and short follow-up time. Further research is necessary to establish the effectiveness of combined therapy. Similarly, one patient who received radiotherapy alone showed no recurrence or progression of the mass after 3 months of follow-up, but more cases and longer follow-up times are needed to determine the efficacy of radiotherapy alone. In patients who do not receive any treatment, nasopharyngeal amyloidoma may progress to systemic amyloidosis or develop into an unresectable mass during long-term follow-up in some cases.

Among the 17 cases with amyloid type mentioned, 9 patients underwent complete tumor resection, 2 patients (1 case of AL type and 1 case of AA type) received chemotherapy combined with surgical resection, and 1 patient with AL type amyloidosis received radiotherapy alone. These twelve patients had no recurrence or progression at known follow-up records. Further research is needed to determine the effects of chemotherapy and radiotherapy for different types of amyloid.

Currently, surgery is the main treatment for nasopharyngeal amyloidoma, but due to the tumor's location, some postoperative patients experience dysfunction. Thus, amyloid typing may be necessary for local amyloidoma, and identifying the type of protein is essential for potential drug treatment, which may lead to a better prognosis for patients with nasopharyngeal amyloidoma. Besides, amyloid typing is also crucial when suspecting systemic disease. In clinical practice, immunohistochemistry and mass spectrometry are the most commonly used methods for detecting amyloid protein types, allowing for accurate identification and quantification of these proteins.

In summary, complete resection of the tumor is essential, and long-term follow-up is necessary after surgery to detect any tumor recurrence or progression in a timely manner, allowing for early treatment. In addition, we need more case evidence and longer follow-up time to verify the effect of surgery combined with chemotherapy or radiotherapy.

## Conclusions

Nasopharyngeal amyloidoma is a rare and distinctive solid tumor. Despite being benign, it is characterized by chronic and aggressive growth and has the potential to progress to systemic amyloidosis, affecting various organs throughout the body. The majority of individuals affected are adults over the age of 40. Early clinical and pathological diagnosis is crucial, and surgical intervention is the primary treatment option for this disease, which typically has a favorable prognosis. Long-term monitoring is necessary to detect potential relapses and initiate timely intervention.

## Data Availability

The data used and/or analyzed during the current study are available from the corresponding author on reasonable request.
